# New insights into honey bee viral and bacterial seasonal infection patterns using third-generation nanopore sequencing on honey bee haemolymph

**DOI:** 10.1186/s13567-024-01382-y

**Published:** 2024-09-27

**Authors:** Cato Van Herzele, Sieglinde Coppens, Nick Vereecke, Sebastiaan Theuns, Dirk C. de Graaf, Hans Nauwynck

**Affiliations:** 1 Department of Translational Physiology, Infectiology and Public Health, Ghent University, Ghent, Belgium; 2 Department of Biochemistry and Microbiology, Ghent University, Ghent, Belgium; 3 PathoSense BV, Pastoriestraat 10, 2500 Lier, Belgium

**Keywords:** Honey bees, third-generation nanopore metagenomic sequencing, epidemiology

## Abstract

Honey bees are rapidly declining, which poses a significant threat to our environment and agriculture industry. These vital insects face a disease complex believed to be caused by a combination of parasites, viruses, pesticides, and nutritional deficiencies. However, the real aetiology is still enigmatic. Due to the conventional analysis methods, we still lack complete insights into the honey bee virome and the presence of pathogenic bacteria. To fill this knowledge gap, we employed third-generation nanopore metagenomic sequencing on honey bee haemolymph to monitor the presence of pathogens over almost a year. This study provides valuable insights into the changes in bacterial and viral loads within honey bee colonies. We identified different pathogens in the honey bee haemolymph, which are not included in honey bee screenings. These pathogens comprise the *Apis mellifera* filamentous virus, Apis rhabdoviruses, and various bacteria such as *Frischella* sp*.* and *Arsenophonus* sp*.* Furthermore, a sharp contrast was observed between young and old bees. Our research proposes that transgenerational immune priming may play a role in shaping infection patterns in honey bees. We observed a significant increase in pathogen loads in the spring, followed by a notable decrease in pathogen presence during the summer and autumn months. However, certain pathogens seem to be able to evade this priming effect, making them particularly intriguing as potential factors contributing to mortality. In the future, we aim to expand our research on honey bee transgenerational immune priming and investigate its potential in natural settings. This knowledge will ultimately enhance honey bee health and decrease colony mortality.

**Supplementary Information:**

The online version contains supplementary material available at 10.1186/s13567-024-01382-y.

## Introduction

Honey bee populations are declining at a concerning rate. This decline has been consistently reported over the last two decades, yet a definitive solution has yet to be identified [1]. Pollinators play a critically important role not only in maintaining the well-being of the environment but also in sustaining the agriculture industry [2]. Honey bees are among the most significant pollinators due to their large colony size and ease of management. Furthermore, they offer a range of products, including honey, pollen, wax for food processing, propolis for use in food technology and medicine, venom in medicine, and royal jelly as a dietary supplement and food ingredient [3]. Alarmingly, these crucial insects are under threat due to a disease complex believed to be caused by a combination of parasites, viruses, pesticides, and nutritional deficiencies [4]. Despite ongoing research, many aspects of this mortality complex remain unknown, primarily for two reasons.

First, the disease complex is intricate because many stress factors work synergistically, and clear symptoms such as hive death may not appear until long after the exposure period [5]. A key reason for winter mortality is that the hive is too weak at the start of winter, either because of the lowered number of bees or their short life span. This reduced number cannot be compensated by more bees being born, as only a small number are born during winter due to the low environmental temperatures. Nevertheless, when the hive eventually dies, it is not certain that any of the abovementioned stress factors still exist. The fate of the hive might have been determined months earlier [6]. For this reason, information on viral infection over a longer period is crucial. Studies frequently only sample at one particular time point, so viral kinetics and their importance in the honey bee mortality complex cannot be determined [7, 8].

Second, the conventional methods of analysis are either outdated or suboptimal. For instance, (RT-)PCR has historically determined viral prevalence. PCR can only detect predetermined or known viruses, so some important viruses can be missed [7, 9–11]. However, recent technical developments, namely third-generation sequencing, have solved this problem. Metagenomic long-read sequencing approaches enable the *ad random* detection of all viruses in a sample. This technique has already been proven vital in identifying new potential contributors to complex diseases in pigs, cattle, horses, and other animals [12–18]. But, it is essential to carefully select the sample type.

Previous studies typically used whole bees, which can lead to interference from unrelated viruses, such as plant viruses that may be present in the bee’s gut and/or on its surface [10]. The use of whole bees is also part of the reason why information on the role of honey bee pathogenic bacteria is scarce. For example, there are only five known pathogenic bacteria in honey bees, which is low compared to other animals such as pigs and cattle [19]. Analysing whole bees makes it difficult to separate bacterial infection from facultative pathogenic or commensal gut bacteria [20].

In addition to the above, other factors need to be considered. For instance, adult honey bees have specific tasks depending on their age and can be divided into two distinct groups: nurses and foragers. Honey bees that are 0 to 2 weeks old are referred to as nurses and perform in-hive tasks, including caring for the brood. Those 2 to approximately 4 weeks old are considered foragers and fly out of the hive to collect food and water [21]. Moreover, there is a unique group of honey bees known as winter bees. These bees start "winter cluster formation", which is usually from October till February, depending on the weather. Few or no new honey bees are born during this period, and the hive temperature drops*.* Winter bees have a longer lifespan of about four months instead of the usual four weeks, as they play a critical role in caring for the upcoming generation in the following spring. Each of these types of bees possesses a unique immune system capacity that can affect their susceptibility to pathogens, resulting in different viral and bacterial loads [15, 22–24]. Winter bees are often overlooked in studies because opening a hive during this season can be harmful to the colony [8]. As a result, the viruses and bacteria present in honey bee hives may not be fully understood, leading to a limited understanding of their impact on honey bee mortality.

This study presents a contemporary approach to researching honey bee colony mortality to address the research gaps identified in previous studies. We followed-up a honey bee apiary to visualise pathogen circulation over time. We sampled honey bees of different ages (from one day old to foragers) to decrease the risk of missing pathogens and to visualise pathogen evolution in certain age groups. We used third-generation nanopore metagenomic sequencing of honey bee haemolymph collected by a newly presented method. This allowed us to identify all pathogens (viruses and bacteria) without contamination from the gut microbiome. To our knowledge, this is the first study in which bacterial and viral loads in the honey bee haemolymph are longitudinally followed up for almost a year.

## Materials and methods

### Honey bee collection

**Figure 1:**
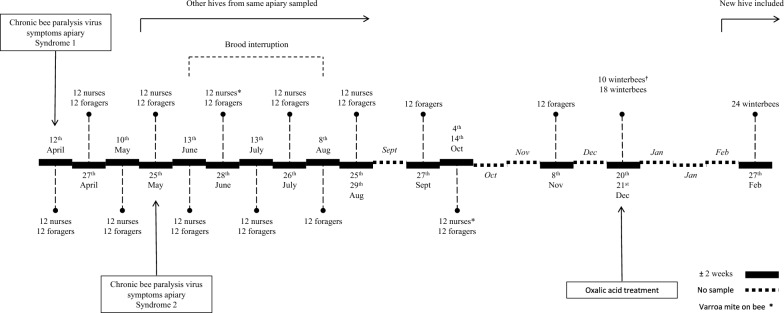
**Timeline honey bee collection.** The bars represent two-week periods. The full bars represent periods during which samples were taken; the dotted bars represent periods when no samples were collected.

A schematic overview of the collected honey bees, hive treatments and disease symptoms can be found in Figure 1. Four beehives at a single apiary were monitored from April 2022 until February 2023. The apiary was situated at Ghent University in Belgium. The sampled bees were a hybrid of *Apis mellifera carnica*, *Apis mellifera Buckfast*, and *Apis mellifera mellifera*. During each visit, three one-day-old nurses were sampled per hive on the brood frames. We opted for taking one-day-old nurses as they can be easily distinguished from older bees—such as forager bees who sometimes walk on the brood frames—by their velvety appearance. Three additional foragers per hive were collected at the hive entrance.

During the honey bee production season, samples were taken every two weeks, with the exception of August and September. From June 14, 2022, to August 1, 2022, the queens were confined for brood interruption, so no young bees were present. By the end of this period, no more brood was present, and an oxalic acid nebulisation was performed to kill the Varroa mites on the adult bees. Due to bad weather in September, only foragers were sampled. Approximately half of the apiary was moved to another location between the first and second sampling of May. Due to unforeseen circumstances, the original four hives could not be sampled further, so four other hives from the same apiary in the new location were chosen for sample collection.

The lifespan of a summer honey bee is approximately four weeks. By sampling nurses and foragers every two weeks, we could profile pathogens in relation to the bee’s age. We only sampled once in October to avoid disturbing the bees while they formed their winter cluster. Only three foragers per hive were taken in November for the same reason. One sample was taken in December, and six winter bees per hive were collected. This was done during the winter Varroa treatment (oxalic acid drip) when the hives had to be opened, so no additional stress was put on the colonies. One hive died before this procedure, after which ten dead bees were collected for further analysis. In February, six bees per hive were sampled at the opening on top of the hive. Again, four hives were sampled, including the three surviving hives and one other hive on the apiary. The samplings took place on 12/04/2022, 27/04/2022, 10/05/2022, 25/05/2022, 13/06/2022, 28/06/2022, 13/07/2022, 26/07/2022, 08/08/2022 (only foragers), 25/08/2022 (foragers), 29/08/2022 (nurses), 27/09/2022 (only foragers), 04/10/2022 (nurses), 14/10/2022 (foragers), 08/11/2022 (foragers), 20/12/2022 (dead winter bees), 21/12/2022 (winter bees) and 27/02/2023 (winter bees).

### Haemolymph collection

The honey bees were sedated for 30 min on ice. Subsequently, the bees were washed with ultrapure water and alcohol to rinse off pollen and sterilise the surface. After drying, the haemolymph was collected using a newly established technique in a class 2 biosafety cabinet. The abdomen was punctured between the second and third tergit with a 24G needle. A sterile anticoagulant (0.5 mL, 54 mM EDTA-PBS, autoclaved) was injected into the thorax using a 30G needle mounted on a 0.5 mL syringe. The anticoagulant haemolymph mixture exited the bee through the previously made puncture wound, resulting in 500 µL in total per bee. Anticoagulant was used to minimise cellular reaction with potential loss of pathogens. Fifty µL of the 500 µL haemolymph mixture from each bee was diluted in 50 µL sterile PBS. Fifty µL of this dilution was pooled per age and sequenced using third-generation nanopore sequencing. The other 50 µL was preserved at −70 °C. The remaining 450 µL of the original haemolymph mixture was used for other research purposes. After the mortality of one hive in December, we analysed the stored frozen haemolymph samples from November separately to see if a different pathogen load could be seen between the surviving hives and the dead hive. The ten dead bees were flushed in the same manner as the live bees. These samples were also pooled and sequenced using third-generation nanopore sequencing.

### Third-generation nanopore metagenomic sequencing

Nanopore metagenomics sequencing was conducted at the PathoSense laboratory, following previously established protocols [12, 18, 25]. These protocols were subjected to quality assessments regarding their sensitivity compared to qPCR [18, 25]. Additionally, the PathoSense laboratory performs frequent validation assays using mock communities. In brief, honey bee haemolymph samples underwent enrichment via filtration using a 0.8 µm polyethersulfone filter (Vivaclear, Sartorius) and centrifugation at 2000 × *g* for 5 min to remove cells and debris. A spike-in virus was introduced to ensure quality control and semi-quantification in downstream data analysis. The filtrates obtained were subjected to nuclease treatment to remove any free nucleic acids and only identify clinically relevant species, excluding any free non-infectious nucleic acids. Subsequently, reverse transcription and *ad random* amplification were performed, as described earlier [12, 25]. The obtained (c)DNA was subjected to rapid library preparation using the SQK-RBK096 library preparation kit (ONT), allowing multiplexing of up to 96 samples per run. No more than 24 pooled haemolymph samples were analysed per run for sequencing throughput. Sequencing was performed on R9.4.1 flow cells (ONT) on a GridION device, facilitating real-time data acquisition, super accurate base calling, and demultiplexing through guppy (v.6.1.5; ONT).

Taxonomic classification of the honey bee associated reads was accomplished using in-house validated databases, with additional validation against the complete National Center for Biotechnology Information (NCBI) database. A semi-quantitative report was generated by comparing the resulting classified read numbers to the reads attributed to the spike-in virus, enabling the categorisation of viral and bacterial loads into five levels: very low, low, medium, high, and very high, as previously determined via comparison with qPCR [25]. For species classification of the bacteria, reporting was limited to the genus level due to sequencing accuracy limitations, as described earlier [18]. Negative PBS controls were included to identify reads that might result from the procedure (i.e., used kits, enzymes, buffers, etc.) and not the bee haemolymph.

#### Viral whole genome sequence analysis

Where sufficient absolute viral reads were obtained in each sample, virus-specific reads were extracted from the complete sequencing output and viral genomes were assembled using canu (v2.2; [26]) and polished with minimap2 (v. 0.2−r123; [27]) and medaka (v1.7.3; ONT), respectively. The genomes were inspected and manually curated. For Deformed wing virus (DWV), all available whole genome sequences were downloaded from NCBI (accessed on 15 May 2023; *n* = 121 respectively and aligned together with our DWV whole genome strains using MAFFT (v.7.453; [28]) prior to phylogenetic inference using IQ-TREE (v1.6.12; [29, 30]). The IQ-tree software allowed the selection of the best substitution model by its built-in modelfinder [31] and was run with 1000 ultrafast bootstrapping trees (–b). Identification of recombination was done using RDP5 using default settings [32]. Recombinants were considered true, employing a conservative approach, if all seven recombination detection methods showed a significant recombination signal. To reconstruct a full phylogenetic tree (model GTR + F + R10) and a reduced tree (model GTR + F + G4), MAFFT and modelfinder (IQ-TREE) were used.

### Statistical analysis

A maximum possible prevalence calculation was performed using WinEpi. We estimated a hive contains approximately 30 000 nurses and 30 000 foragers during bee season, which results in a maximum possible prevalence of 41.52% per colony (three bees sampled) when the pooled sample is negative. In winter, we took six winter bees with 10 000 bees per hive, which results in a maximum possible prevalence of 23.53% when the pooled sample is negative. The confidence level was set at 80%.

## Results

**Figure 2:**
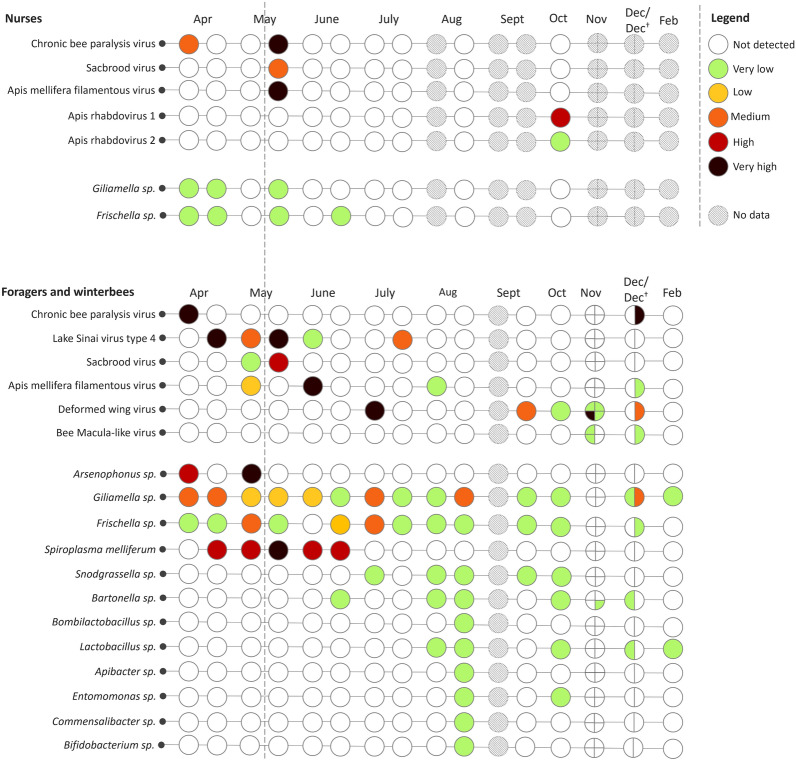
**Overview of detected viruses and bacteria.** Third-generation nanopore sequencing output of nurses, foragers, live winter bees (Dec and Feb) and dead winter bees (Dec†). In November, the four honey bee hives were analysed separately, starting from the upper left and moving clockwise. A semi-quantitative report was generated by comparing the data to the spike-in virus previously included, enabling the categorisation of viral and bacterial loads into five levels: very low, low, medium, high, and very high. The dashed line indicates the point from which different hives from the same apiary were used.

During the entire sampling period, several viruses and bacteria were detected. For this discussion, we will only focus on the bacteria and viruses associated with honey bees, as shown in Figure 2. The excluded viruses and bacteria were associated with plants such as Cherry leaf roll virus or were kitome associated such as *Acinetobacter* sp.*.* Raw read sequencing output is uploaded to the Sequence Read Archive (SRA).

Eight different honey bee viruses were detected, including Chronic bee paralysis virus (CBPV), Lake Sinai virus type 4 (LSV-4), Sacbrood virus (SBV), *Apis mellifera* filamentous virus (AmFV), Deformed wing virus (DWV), Apis rhabdovirus 1 (ARV-1), Apis rhabdovirus 2 (ARV-2) and Bee Macula-like virus (BMLV). While CBPV, LSV-4, SBV, AmFV and DWV were found in foragers, nurses showed the presence of CBPV, SBV, AmFV, ARV-1 and ARV-2.

The dead bees were found to carry several viruses, such as CBPV, AmFV, DWV, and BMLV. CBPV was detected in spring and autumn; LSV-4 and SBV in spring; AmFV in spring, summer, and autumn; DWV in summer and autumn; ARV-1, ARV-2 and BMLV in autumn. A pairwise sequence analysis was performed for LSV-4 as three complete genomes could be reconstructed. The LSV-4 viral genomes in the May sample showed a pairwise sequence identity of 99.80% compared to the first detection in April. A further drop to 97.59% was seen in the sample from July.

**Figure 3:**
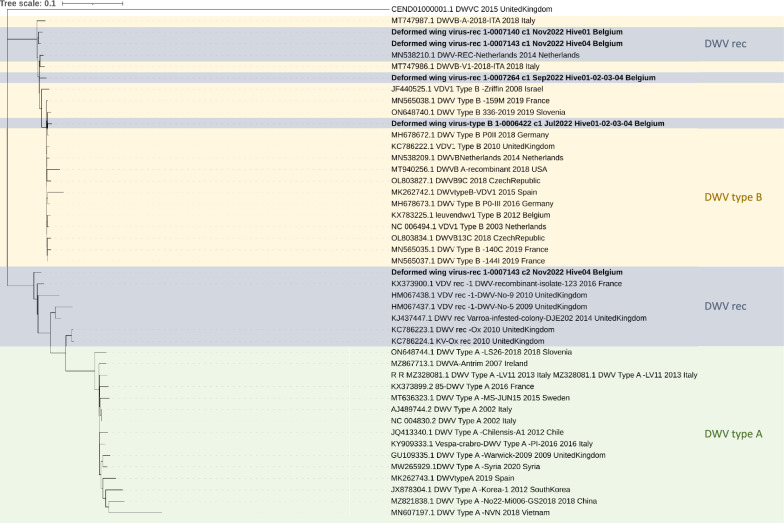
**Reduced phylogenetic tree deformed wing virus.** Phylogenetic analysis (GTR + F + G4) shows a genetic distinction between the samples taken at different time points: DWV-B in July (Deformed wing virus-type B/1-0006422_c1_Jul2022_Hive01-02–03-04 Belgium), DWV-rec in September (Deformed wing virus-rec/1-0007264_c1_Sep2022_Hive01-02–03-04 Belgium), DWV-rec in hive 1 (Deformed wing virus-rec/1-0007140_c1_Nov2022_Hive01 Belgium) and 2 types of DWV-rec in hive 4 (Deformed wing virus-rec/1-0007143_c1_Nov2022_Hive04 Belgium and Deformed wing virus-rec/1-0007143_c2_Nov2022_Hive04 Belgium) in November, shown in bold. DWV-rec is a recombinant between DWV-A and DWV-B. The DWV type B clade is shown in yellow, DWV type A is shown in green, and DWV-rec is shown in blue.

Phylogenetic analyses were conducted using the full genome sequences to determine the relationship between the new viral sequences found in our samples and those publicly available on NCBI. The study’s discussion will focus solely on the phylogenetic tree of DWV due to the significant variability observed among samples. The full genome sequences were uploaded to the NCBI GenBank, and their accession number can be found in Additional file 1, while the full phylogenetic tree of DWV can be found in Additional file 2. All sequences clustered into two distinct clades called "DWV" (or Type A) and "VDV-1" (or Type B) (see Figure 3, reduced tree). Additionally, recombinants between Types A and B were found.

An RDP analysis was performed to verify if recombination was present in the DWV strains. The DWV viral types were DWV-B in July, DWV-rec (recombinant between DWV-A and DWV-B) in September, and DWV-rec in hive 1 and 2 types of DWV-rec in hive 4 in November (c1 and c2). The absolute DWV viral loads in the sampling of October, the sampling of hives 2 and 3 in November and the dead hive were too low to obtain viral genome assemblies for these DWV strains.

In addition to the detection of viruses, we also detected 12 different bacterial species: *Arsenophonus* sp*.*, *Gilliamella* sp*.*, *Frischella* sp*.*, *Spiroplasma melliferum*, *Snodgrassella* sp*.*, *Bartonella* sp*., Bombilactobacillus* sp*., Lactobacillus* sp., *Apibacter* sp., *Entomomonas* sp., *Commensalibacter* sp. and *Bifidobacterium* sp. *Giliamella* sp. and *Frischella* sp. were detected in both nurses and foragers. All other genera were detected solely in foragers. *Giliamella* sp., *Bartonella* sp., and *Lactobacillus* sp. were also detected in winter bees. In dead winter bees, *Giliamella* sp. and *Frischella* sp. were detected. *Arsenophonus* sp. and *Spiroplasma melliferum* were detected in spring and summer; *Snodgrassella* sp. *and Bartonella* sp. were detected in summer and autumn; *Bombilactobacillus* sp., *Apibacter* sp., *Commensalibacter* sp., and *Bifidobacterium* sp. in summer; *Lactobacillus* sp. in summer, autumn, and winter; *Entomomonas* sp. and *Snodgrassella* sp. in summer and autumn; *Giliamella* sp. in spring, summer, autumn, and winter; *Frischella* sp. in spring, summer, and autumn.

Overall, fewer viral and bacterial species and loads were detected in nurses than foragers. Nurses had their highest pathogen load in the spring, while foragers had a high pathogen load in the spring and at the beginning of summer. In autumn and winter, the pathogen load was lower but more diverse. In live winter bees, the pathogen diversity was low.

## Discussion

In this study, we conducted an extensive analysis using third-generation metagenomic nanopore sequencing on forager and nurse haemolymph samples spanning nearly a year. We found that nurses and foragers show different viral and bacterial loads and diversity. This is unsurprising as nurses and foragers encounter different pathogens due to their distinctive hive tasks. Furthermore, it is important to note that foragers and nurses possess varying immunities, including differing haemocyte numbers and compositions. These differences may contribute to variations in susceptibility to pathogens and their resulting effects on disease outcomes [22, 23]. Although variations in pathogens were expected, most studies do not differentiate between different age groups of bees or mention the age of the bees [7, 9, 11]. This lack of differentiation in honey bee samples can lead to markedly divergent outcomes and conclusions on apiary health. Our findings serve as a demonstration of this phenomenon.

A total of eight viruses were detected, with Table 1 providing an overview of their classification, symptoms, and proven transmission routes. Notably, identifying only eight viruses sharply contrasts the 30 viruses identified in Gebremedhn et al. [10], where they similarly employed next-generation sequencing to explore the honey bee virome. This divergence may be attributed to their sampling of whole bees, which possibly introduced a significant number of contaminating viruses. Furthermore, the study explicitly noted that of the 30 viruses found, only five were specific to honey bees, 15 were specific to plants, and the remaining 10 were specific to insects. We were able to detect four of the five viruses from the Gebremedhn et al. study [10], namely DWV, SBV, AmFV, and LSV, which stresses the added value of using haemolymph as a sample.

**Table 1:** **Overview identified viruses**

Virus	Family genome	Symptoms	Transmission proven
Chronic bee paralysis virus	Unclassified Enveloped ssRNA( +)	Syndrome 1: trembling bees with a bloated abdomen. These bees are unable to fly; they crawl in front of the hive and die within a few days Syndrome 2: hairless and darker bees (called black robbers) who suffered nibbling attacks by healthy bees. They also die within a few days [8, 34]	Oro-faecal [34] Contact [79]
Lake Sinai virus	Unclassified ssRNA( +)	Associated with bad colony health, but symptoms are unknown [37]	Unknown
Sacbrood virus	Iflaviridae non-enveloped ssRNA( +)	Causes pupation failure, which results in swollen larvae filled with fluid. Young infected adult bees show precocious foraging and impaired foraging activity with a reduction of life span. Older adults can be infected but are asymptomatic [8] Pupae can also be asymptomatic [80]	Oro-faecal [8]
*Apis mellifera* filamentous virus	Baculoviridae Enveloped dsDNA	Milky-white haemolymph [8, 50]	Oro-faecal [50]
Deformed wing virus	Iflaviridae non-enveloped ssRNA( +)	Causes crumpled or aborted wings, shortened abdomens, paralysis, severely shortened adult life span, impaired learning, and foraging behaviour [8, 47]	Oro-faecal [47] Vertical [47] Varroa [8, 47]
Apis rhabdovirus 1 and 2	Rhabdoviridae Enveloped ssRNA(−)	Unknown	Unknown
Bee macula-like virus	Tymoviridae ssRNA( +)	Unknown	Unknown

Overview of the identified viruses, their classification (family and genome), the known symptoms and proven transmission routes

The first virus detected was the Chronic bee paralysis virus (CBPV). At the beginning of our observations in April, one hive in the apiary displayed clear clinical signs of CBPV (syndrome 1), but the hives included in this sampling did not. Although no visible symptoms were found in the sampled hives, we detected the virus in both the nurses and foragers during the first sampling, consistent with previous research [33]. The virus was no longer detected during the second sampling in April and the following sampling in May. After the first sampling in May, the honey bee hives were moved. One newly included hive displayed clear CBPV symptoms in April.

CBPV was detected again during the second May sampling in nurses, with syndrome 2 present in one of the sampled hives. This may have been due to the reintroduction of the virus from bees of neighbouring apiaries or other insects [34, 35]. It is also possible that the move induced stress, which can lower the immune system and result in more individuals becoming infected [36, 37]. CBPV was found to be present in both nurses and foragers, which is not surprising as CBPV can be transmitted horizontally and presumably also vertically [34, 38]. The virus was no longer found two weeks after the nurses were initially detected to have CBPV. This finding could be due to multiple reasons, such as an immune response in the adults (mainly RNA interference), mortality or removal of the infected bees, behavioural responses such as fever, the number of infected individuals or viral levels below our detection limit), or a non-homogenous viral spread in the population [39].

Nevertheless, this pattern of CBPV infection is consistent with previous research that indicates the virus is predominantly present in the spring and summer months [8]. In December, CBPV was once more detected in the dead hive, suggesting that the virus could have been important in the downfall of this particular hive, especially as CBPV has been linked with winter mortality before [40]. In winter, honey bees are more susceptible to viral diseases as they experience an immune depression [41]. CBPV is considered an important emerging disease, particularly as cases increase exponentially over time, although outbreaks are region-dependent [42]. Several Belgian screenings have been conducted, which reveal that CBPV was detected in 29% of the colonies in 2017. This number demonstrates an increase compared to the 1.7% found in Flanders in 2011 but a considerable decrease compared to the 69% found in Wallonia in 2006 [7, 43].

The second virus, Lake Sinai virus type 4 (LSV-4), was detected from April to June and again in July. LSV-4 was first detected in Belgian forager bees in 2011 [44]. Interestingly, we only detected LSV-4 in foragers and not in nurses. It is suggested that Varroa mites play a role in transmitting this virus, but this is still under debate [8, 45, 46].

The Sacbrood virus (SBV) was detected in May in both foragers and nurses without apparent symptoms. Previously, asymptomatic infections of SBV have been reported [8]. Almost all hives were positive during a Belgian screening in the spring of 2017. This finding contrasts with a previous study in July 2011, where only 19% of hives tested positive [7, 43]. According to earlier research, SBV is mostly present in spring, corresponding with our results [7, 8, 43].

We found that the Deformed wing virus (DWV) circulated in live bees in June, and September through October. Interestingly, DWV was detected exclusively in foragers, although some honey bee nurses (second sampling of June and October samplings) were found to be carrying Varroa mites. DWV-B, the most prevalent genotype, is transmitted mainly via Varroa mites that feed on honey bee pupae [8, 47]. We noted that the sampled nurses did not show deformed wings, so the viral load may have been too low [48]. Another possibility is that the Varroa mites present on the nurses did not carry DWV-B. A recent study found that approximately only 40% of collected mites were able to induce a high (overt) level DWV infection [49]. As mentioned, DWV is a prevalent virus, demonstrated in a previous Belgian study from the spring of 2017 when all the screened Belgian hives tested positive [7]. In the 2011 Belgian screening, DWV-A and DWV-B were not differentiated, but the overall DWV prevalence was still high at 69% [43]. Furthermore, DWV was also detected in the dead bees. In literature, DWV has also been associated with winter mortality [8, 34, 47].

*Apis mellifera* filamentous virus (AmFV) was detected in live bees in May, June, and August. This result corresponds to previous studies that detected AmFV all year round [50]. However, AmFV was detected in both foragers and nurses, which contradicts the results of Hartmann et al. [50], where it was reported that worker bees became infected one week post-emergence [50]. No impact on overwintering and AmFV has been found [50], although we identified AmFV in the dead hive.

Apis rhabdovirus 1 and 2 (ARV-1 and ARV-2), sometimes called bee rhabdoviruses, were detected in October [51]. Thanks to next-generation sequencing, ARV has only recently been identified, so very little is known about them [11]. We found that only the nurses were infected with ARV, indicating that the infection occurred before they emerged. ARV was not detected in foragers, which suggests that either the nurses died before they could start foraging (which is, for example, the case in CBPV infection and the so-called ‘black robbers’), or they were able to control viral replication and eliminate the virus.

The Bee Macula-like virus (BMLV), also known as Varroa destructor Macula-like Virus, was discovered in November in two surviving hives and in the dead hive in December. In July 2011, BMLV was detected in 84% of Belgian colonies, but no correlation with colony mortality was found [44].

We also detected a wide range of bacteria in addition to viruses. We propose different ways these bacteria could be detected in the haemolymph, including pathogenic bacteria that colonise it, intestinal leakage, Varroa mite bacterial injection via feeding, and the physiological haemolymph microbiome.

Some bacteria are considered pathogenic and colonise the haemolymph, for example, by breaching the gut barrier. We identified *Spiroplasma melliferum,* a well-known honey bee pathogenic bacterium*. Spiroplasma* sp. are presumed to be the causative agents of May disease or spiroplasmosis. The symptoms of May disease include bees that appear to be crawling and trembling, with a swollen and hard abdomen caused by the accumulation of undigested pollen in their gut. Although individual bees can die, colonies recover naturally. It is presumed that *Spiroplasma* sp. breaches the midgut barrier to colonise the haemolymph [52]. No symptoms of May disease were noted in our hives, but the bacteria were detected in spring, which is the typical period during which this disease occurs. In July 2011, a Belgian screening found a *Spiroplasma melliferum* presence of 4.4% without an association with winter mortality [44]. It is possible that some of the other detected bacteria could be pathogenic, but they have not yet been linked to honey bee disease. Currently, only five pathogenic bacteria have been described [19].

*Frischella perrara *causes scab formation, which is a melanin deposit at the end of the honey bee midgut called the pylorus. Furthermore, the ‘scab’ can be colonised by other bacteria, such as *Giliamella apicola* and *Snodgrassella alvi* [53]. This melanin production is an immune response that may help prime newly emerged workers, as suggested in previous studies [54]. We suggest that this bacterium can penetrate the gut barrier by suppressing the immune system and entering the haemolymph. Engel et al. found that scab formation is absent in newly emerged nurses but develops in older nurses [53]. This finding is noteworthy as, in our study, *Frischella* sp. were detected in nurse haemolymph. It is possible that these nurses did not have the immune capacity to form a melanin plaque and, therefore, could not stop the penetration of this bacterium.

One potential cause of this immune suppression could be the stress caused by the translocation of the hives in May. Thus, the prevalence of *Frischella* sp. in honey bee haemolymph could be important and require further investigation. Additionally, physiological or pathological intestinal leakage of gut microbiome bacteria could result in bacterial detection in the haemolymph. Some identified bacteria are part of the honey bee gut microbiome, such as *Arsenophonus* sp., *Giliamella* sp., *Frischella* sp., *Snodgrassella* sp., *Bartonella* sp., *Bombilactobacillus* sp., *Lactobacillus* sp., *Apibacter* sp., *Commensalibacter* sp. and *Bifidobacterium* sp. [55–58]. It is possible that other detected bacteria are also part of the gut microbiome but have not yet been identified in previous studies. For example, *Entomomonas* sp. was detected only recently (in 2020) in Asian honey bees [59].

Moreover, oxalic acid treatment can potentially produce intestinal leakage as it has been proven to cause honey bee mortality, intestinal cell death, and keratin destruction [60–62]. The hives in this study were treated with oxalic acid at the start of August, but the bacterial species did not reach high loads. However, a wider variety of species were detected. Furthermore, Varroa mites can directly inject bacteria into the haemolymph [63]. In some insects, the existence of a haemolymph microbiome has also been described [64]. Nevertheless, the fact that these bacteria can enter the haemolymph highlights the need for more research on bacterial infections in honey bees.

Notably, the viral and bacterial load was significantly higher during the spring and early summer, as several other studies have also observed [8, 33, 50, 65, 66]. This finding is surprising because there is always a constant risk of reintroducing viruses and bacteria from other apiaries or insects [34, 67, 68]. This reintroduction can also be seen in our results as AmFV and LSV-4 reappear later in the season in live honeybees. Another possibility is that these two viruses were still present in a small number of bees, but the hive could no longer control them due to factors such as immune depression caused by brood interruption [35]. Nevertheless, when a virus is detected again in the bee hives in this study, they do not reach high loads. However, newly introduced viruses, such as ARV, do reach higher values. This pattern suggests that the bees can protect the new generation from infection, similar to a phenomenon called trans-generational immune priming (TGIP) observed in honey bees. TGIP functions at the colony-wide level to safeguard the next generation [69]. The primary focus of TGIP research in honey bees has been on bacterial infections, such as *Paenibacillus larvae*, the causative agent of American foulbrood. These findings have led to the recent U.S.D.A. approval of the first honey bee ‘vaccine’ against American foulbrood [70, 71]. Research on TGIP against viral infections in honey bees has primarily focused on a transmissible RNA pathway due to honey bees defending themselves against viral infection via RNA interference (RNAi), for which dsRNA is the trigger.

There are two exceptions to this pattern: DWV and LSV-4. Although DWV was initially detected in July, it was also found again from September to December. This may be attributed to the introduction of a different genotype. While genotype B (DWV-B) is the most prevalent in Belgium, genotype A (DWV-A) is also present [7]. There have also been numerous reports of recombinants of these two types (DWV-rec) in Europe and other parts of the world [72, 73]. A phylogenetic analysis was performed to verify genotype differences, which showed a clear genetic distinction between the samples taken at different time points (see Figure 3). This difference in genotype can explain why DWV was able to re-infect the hives, which was already proven for different DWV strains [74].

LSV-4 was detected again in foragers in July. However, we noted that the virus had evolved when viral genomes were compared. The pairwise sequence identity of the isolate from May dropped to 99.80% compared to the first detection in April. An even further drop to 97.59% was seen in the sample from July, illustrating the rapid mutation rate and how ssRNA viruses are extremely susceptible to mutations. This mutation process could lead to immune evasion and explain the virus’s reappearance [74]. Recombinant analysis showed no evidence of recombination in any of the three genomes. Other studies that noted a peak in spring and summer, have also reported exceptions. However, it is challenging to determine whether recombination or mutations were present as these studies either used qPCR or did not provide whole genome analyses [33, 37, 65, 66].

Based on our findings, we hypothesise that honey bee colonies become infected with a wide range of pathogens during the spring season, enabling the hive to build up an immunity against these pathogens and to provide protection for themselves and future generations throughout the year, particularly during the crucial winter period. However, when an immune evasive or emerging pathogen emerges during summer or autumn, the acquired immunity is ineffective, leading to infection within the hive. While the bees can overcome less pathogenic pathogens, the hive ultimately collapses if the pathogen is highly pathogenic and/or the immune system is compromised (such as during the winter period or due to genetic factors).

In the study conducted by Truong et al., sick apiaries were analysed, and viruses were detected throughout the year [65]. However, a significant increase was observed in July. This finding aligns with our hypothesis: if immune priming fails, weak colonies will struggle to manage viral infections. One example of how a pathogen can enter an apiary or reach high levels during autumn is when beekeepers move hives or materials from one apiary to another. Another reason is due to insufficient control of the Varroa mite. Honey bees are typically treated against Varroa mites in December or January, but a treatment in autumn could be potentially more beneficial. Understanding the mechanism of TGIP in honey bees is crucial for advising beekeepers and developing strategies to combat viral infections. This can involve implementing measures such as a vaccination which has been used for *Paenibacillus larvae,* genetic selection, or feed additives [70, 71, 75]. We will study this hypothetical model in more detail in future studies.

It is important to note that we did not identify all viruses that were present in the hives. For instance, ARV-1 might have infected the colony prior to our detection and possibly reached elevated levels due to factors such as immune suppression rather than a lack of immune priming. A disadvantage of using haemolymph is the labour-intensive sampling process compared to analysing whole bees, resulting in a smaller sampling size and a higher chance of missing viral infections. It is also essential to acknowledge the limited understanding of infection transmission within a colony [8]. It is unclear whether infection consistently results in a colony-wide spread or if individual and social immunity can effectively eliminate it, particularly given bees’ close contact, such as through trophallaxis. In this study, we set out to determine which pathogens were most abundant in an apiary, because we question the relevance of pathogens found in low abundance or those that could be easily eliminated through individual or social immunity. Furthermore, an important distinction must be made between qPCR on whole bees and third-generation nanopore sequencing on haemolymph. Using haemolymph, we could detect active viral infections, as viruses must breach the gut or exoskeleton barrier to enter the hemocoel without contamination and interference with the gut microbiome.

Our enrichment protocol enables us to exclusively detect active viral infections by eliminating any extraneous free genetic material. QPCR on whole bees can result in the detection of viruses or loose viral genomic material from the surface and gut, which will not, per se, result in infection as high oral dosages are sometimes necessary for viral infection [9]. Extrapolating our data to literature qPCR becomes challenging due to this issue. Although, analysing haemolymph instead of whole bees has a lot of advantages. One disadvantage is that viruses which replicate locally can be missed. Due to the limited understanding of most honey bee viral pathogeneses, these viruses are still unknown [38]. In the study of D’Alvise et al., the Black queen cell virus (BQCV) was present all year round, with a peak in spring and summer [33]. BQCV is suspected to replicate in the gut. In artificially infected queens, it has been identified in 100% of gut samples and 70% of ovary samples but not in the haemolymph, head, spermatheca, and eviscerated body [38]. A potential problem with nanopore sequencing is the possibility that some viruses are abundantly present in the haemolymph after infection compared to others. In this way, it may suppress the detection of low-abundant viruses. Third-generation nanopore sequencing generally exhibits a sensitivity comparable to, but slightly lower than, qPCR, although our enrichment protocol and sample selection have largely mitigated this discrepancy [18, 25]. We did not correlate third-generation nanopore sequencing on haemolymph with qPCR on whole bees as the results would be virus-dependent. Additionally, some novel viruses were identified so correlations are even more challenging.

Furthermore, comparing haemolymph to whole bees is challenging as analysis cannot be undertaken for both in the same bee. When haemolymph is collected, a potentially important viral reservoir is removed; thus, the bee will no longer be representative as a whole bee. The previously mentioned advantages and disadvantages of a small sampling size, haemolymph sampling and third-generation sequencing may explain in part why, in the studies of D’Alvise et al. [33] and Faurot-Daniels et al. [37], viruses could be found all year round in contrast to this study’s results.

In addition to our hypothetical model, other hypotheses for this pathogen peak are also possible. For example, higher honey bee activity and brood density are present during the flowering period (spring and summer). A higher number of individuals, both inside and outside the hive (e.g. contact with other bees), could increase viral transmission, resulting in a viral peak [76]. Another possibility is that Varroa mite infestation could be causing bees to drift into neighbouring hives and spread the infection. However, Varroa mite loads are expected to be highest during autumn [65]. An alternative possibility is the ‘robbing’ of neighbouring hives, most common in late summer to early fall. Other probabilities could be more virus-specific. For example, temperature fluctuations, which are typical in spring, are hypothesised to cause a higher prevalence of SBV [77]. Lastly, this study focused on viruses and bacteria, but other pathogens or chemicals could influence this pattern, such as Nosema sp. and pesticides [78]. For future projects, we intend to incorporate Nosema sp. and chemical analysis to deepen our understanding of this phenomenon.

In conclusion, this study shows the added value of third-generation nanopore sequencing on honey bee haemolymph as AmFV, ARV-1, ARV-2 and a wide variety of bacteria were detected, which are typically not included in studies or screenings. Conducting multiple samplings per year on honey bees of different ages is vital as certain pathogens may not be present consistently throughout the year or in both young and old bees. Finally, the potential role of TGIP under natural conditions was proposed and acknowledged, which will lead to further research. In the future, more apiaries will be followed longitudinally to validate our working hypothesis. Characterisation of the virome and bacteriome present in honey bee haemolymph in function of time will allow us to study which pathogens are causing honey bee hive collapse. Ultimately, this will lead to better overall honey bee health and lower colony mortality.

## Supplementary Information

**Additional file 1. Full genome sequences GenBank accession**. Due to the error rate of Nanopore data, erroneous insertions or deletions may persist within genomes, particularly in regions characterised by low coverage, homopolymers, and repeats. A final manual curation is performed to ensure these are removed. The genome is aligned to the closest NCBI reference, and all inserts and deletions are checked and corrected if necessary. An overview of the viral genomes and their GenBank accession codes can be found in the additional file.

**Additional file 2. Full deformed wing virus phylogenetic tree.** The phylogenetic analysis (GTR + F + I + R10) shows a genetic distinction between the samples taken at different time points: DWV-B in July (Deformed wing virus-type B/1-0006422_c1_Jul2022_Hive01-02-03-04 Belgium), DWV-rec in September (Deformed wing virus-rec/1-0007264_c1_Sep2022_Hive01-02-03-04 Belgium), DWV-rec in hive 1 (Deformed wing virus-rec/1-0007140_c1_Nov2022_Hive01 Belgium) and 2 types of DWV-rec in hive 4 (Deformed wing virus-rec/1-0007143_c1_Nov2022_Hive04 Belgium and Deformed wing virus-rec/1-0007143_c2_Nov2022_Hive04 Belgium) in November, shown in bold. DWV-rec is a recombinant between DWV-A and DWV-B. The DWV type B clade is shown in yellow, DWV type A in green and DWV-rec in blue.

## Data Availability

All data generated or analysed during this study are included in this published article [and its supplementary information files]. The full genome sequences and the raw sequencing output were also deposited into the NCBI database (See Additional file 1—Full genome sequences GenBank accession). SRA BioProject: PRJNA1042938.
