# Supplementary figures and images for: New insights into honey bee viral and bacterial seasonal infection patterns using third-generation nanopore sequencing on honey bee haemolymph
Source: Vet Res. 2024 Sep 27;55:118. doi: 10.1186/s13567-024-01382-y (PMC11430211; doi:10.1186/s13567-024-01382-y)

DWV type A

DWV type B

DWV rec

DWV rec


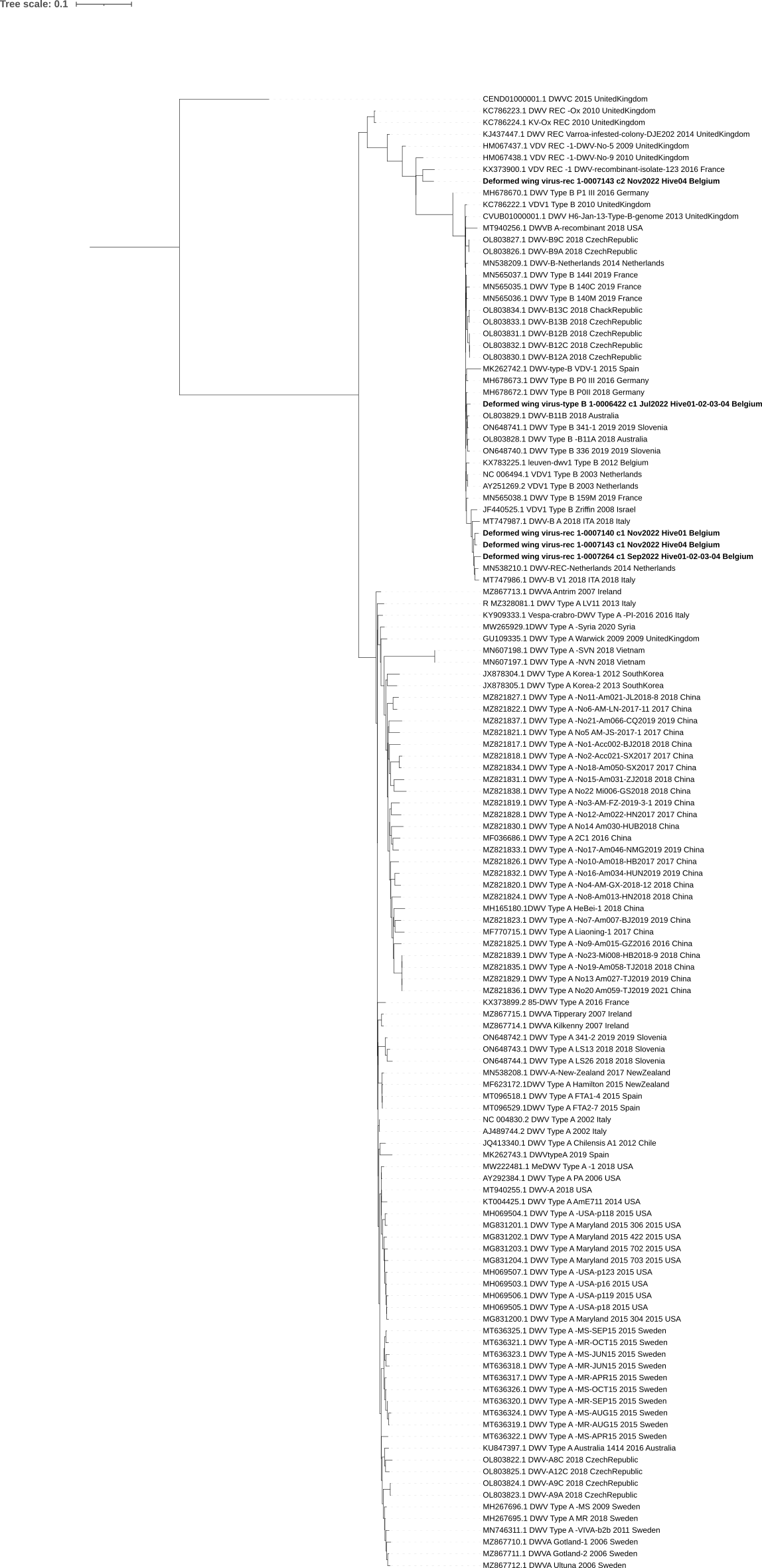

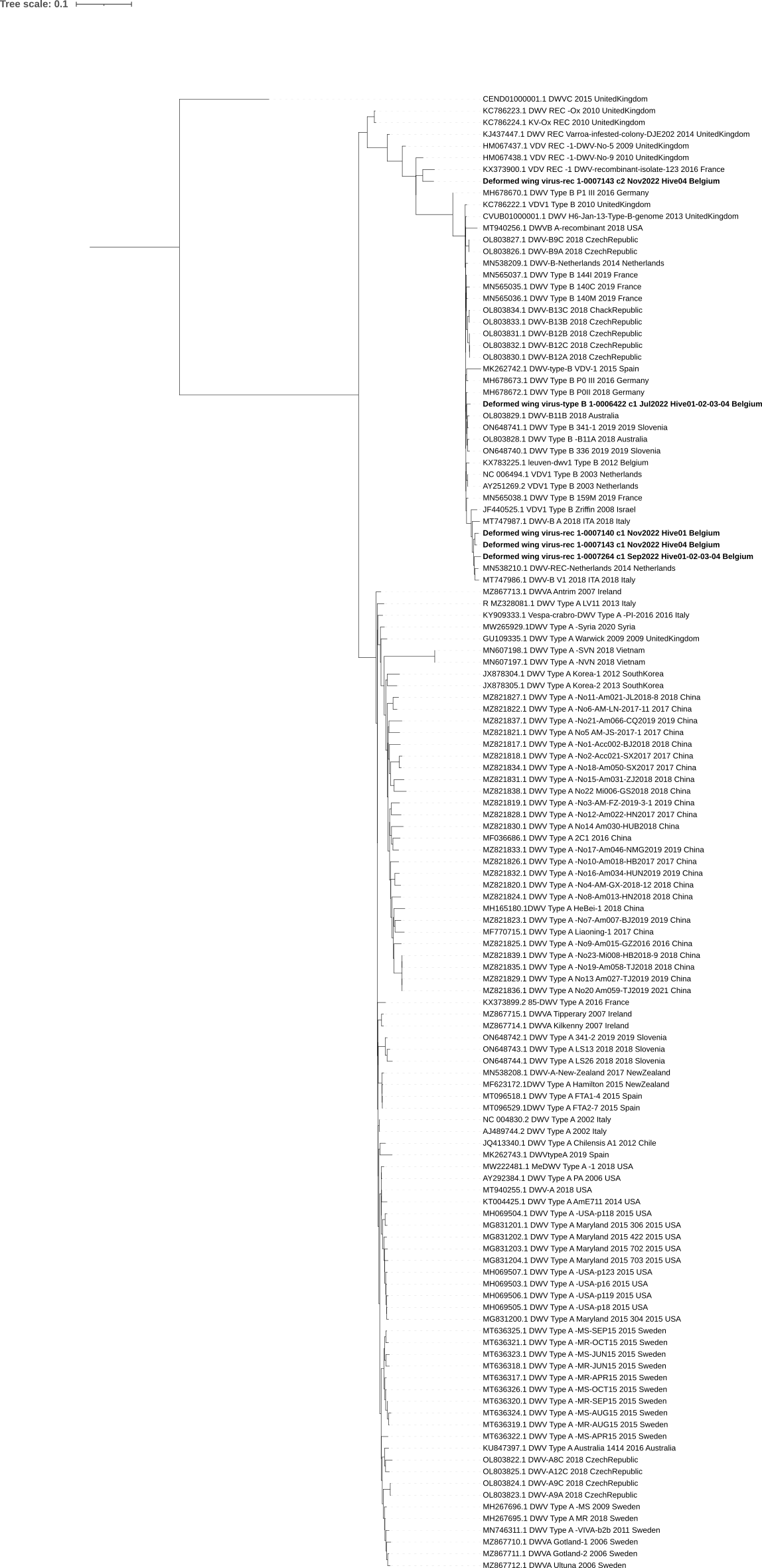

Supplement: Supplementary file 2 — Additional file 2. Full deformed wing virus phylogenetic tree. The phylogenetic analysis (GTR + F + I + R10) shows a genetic distinction between the samples taken at different time points: DWV-B in July (Deformed wing virus-type B/1-0006422_c1_Jul2022_Hive01-02-03-04 Belgium), DWV-rec in September (Deformed wing virus-rec/1-0007264_c1_Sep2022_Hive01-02-03-04 Belgium), DWV-rec in hive 1 (Deformed wing virus-rec/1-0007140_c1_Nov2022_Hive01 Belgium) and 2 types of DWV-rec in hive 4 (Deformed wing virus-rec/1-0007143_c1_Nov2022_Hive04 Belgium and Deformed wing virus-rec/1-0007143_c2_Nov2022_Hive04 Belgium) in November, shown in bold. DWV-rec is a recombinant between DWV-A and DWV-B. The DWV type B clade is shown in yellow, DWV type A in green and DWV-rec in blue. [file 13567_2024_1382_MOESM2_ESM.docx]
